# Supplementary material for: Prevalence and mechanisms of anterior cruciate ligament tears in military personnel: A cross-sectional study in Iran
Source: PLoS One. 2024 Jun 21;19(6):e0303326. doi: 10.1371/journal.pone.0303326 (PMC11192309; doi:10.1371/journal.pone.0303326)
Supplement: Attachment — Submitted filename: ReviewerIran.docx [file pone.0303326.s002.docx]

Reviewer’s Comments

**Anterior Cruciate Ligament Tear in the Iranian Military Setting; the Injury Prevalence and Mechanism**

**Comments for the Author**

Line 22 The introduction lacks a scientific rationale for this study.

Line 23 The author should provide a specific hypothesis.

Line 33 Could all the patients in this study recall the mechanism of injury? E.g., Fall with the knee in valgus position.

Line 35 Difference between military exercises and athletic exercise?

Line 42 Could you please elaborate the prevention strategies this study has helped to develop?

Line 82 Please mention the rationale for excluding the chronic injury.

Line 85 Clinical and imaging findings for ACL tear have inter- observer and intra- observer	variability. If only those patients taken for arthroscopic surgery were studied and	intraoperative findings considered the gold standard, the results would be more	reproducible.

Line 98 Clinical and imaging findings with respect to chondral and meniscus injuries have inter-	observer variability.

Line 128 Please define the military exercise.

Line 176 Please clarify the similarities and differences between the military and sports activities.

Line 216 This study does not determine the exact measures to prevent an injury to ACL.

Line 192 The author has assumed that none of the patients were exposed to warm-up	exercises.

Line 194 A gender sub-analysis could give a better insight.

Line 194 Besides the mechanism of injury, there are other anatomical factors responsible for	ACL injury. Please clarify how these factors have been matched.

Earlier studies have concluded even more detailed mechanisms of injury and prevention strategies. Please elaborate what this study adds extra to the existing knowledge related to the topic.
