# Supplementary material for: Prevalence and mechanisms of anterior cruciate ligament tears in military personnel: A cross-sectional study in Iran
Source: PLoS One. 2024 Jun 21;19(6):e0303326. doi: 10.1371/journal.pone.0303326 (PMC11192309; doi:10.1371/journal.pone.0303326)
Supplement: Attachment — Submitted filename: reply to comments.docx [file pone.0303326.s003.docx]

**Dear Editor,**

**Thank you for evaluating my work. The following comments have been check and edited in the manuscript file.**

Line 22 the introduction lacks a scientific rationale for this study. Edited
Line 23 The author should provide a specific hypothesis. Edited
Line 33 Could all the patients in this study recall the mechanism of injury? E.g., Fall with the knee in valgus position. Yes, as our sample included acute and new cases with knee injury they could recall.
Line 35 Difference between military exercises and athletic exercise? Added and Defined in the methods
Line 42 Could you please elaborate the prevention strategies this study has helped to develop? As mentioned in discussion and conclusion: The findings on mechanisms and prevalence of injury may help develop ACL injury prevention programs that may minimize the occurrence of such injuries in the military setting.
Line 82 Please mention the rationale for excluding the chronic injury. The most important rationale was the recall bias which may occur with a longer time since injury. Other reasons include narrowing diagnosis and associated injuries.
Line 85 Clinical and imaging findings for ACL tear have inter- observer and intra- observer variability. If only those patients taken for arthroscopic surgery were studied and intraoperative findings considered the gold standard, the results would be more reproducible. Yes, right, we did all investigation by one person to reduce any bias and error but included all injured knees in the study.
Line 98 Clinical and imaging findings with respect to chondral and meniscus injuries have inter- observer variability. This also had been investigated by the same person.
Line 128 Please define the military exercise. Defined previously
Line 176 Please clarify the similarities and differences between the military and sports activities. As mentioned, both activities-as mentioned the examples- are the same in terms of magnitude of activity the difference are in kind of activity like combat and sport activities ,etc.
Line 216 This study does not determine the exact measures to prevent an injury to ACL. as we discussed, our study provided data on knowing mechanism and prevalence of the injury which could be taken into account for neutralizing the associated factors of injury or decrease chance of getting injured.
Line 192 The author has assumed that none of the patients were exposed to warm-up exercises.no we mean that although participants will get involve in warm-up exercise before actual activity, at the beginning of the actual and main activity, there would be a greater chance of injury as the body system has just faced real and serious activity, physiologically.
Line 194 A gender sub-analysis could give a better insight. Yes, it was better if more extensive literature were available. we tried to elaborate it anyway.
Line 194 Besides the mechanism of injury, there are other anatomical factors responsible for ACL injury. Please clarify how these factors have been matched. Actually our study was a cross sectional analytic study and we did not do any classic matching.

Earlier studies have concluded even more detailed mechanisms of injury and prevention strategies. Please elaborate what this study adds extra to the existing knowledge related to the topic. We conducted the study in a referral military Iranian hospital which no previous data have been reported.so geographically; our data will help know regional data.

Reviewer #2: Authors should be congratulated for such a nice work. It definitely has scientific merit and will be an interesting topic for readers. However, here are some suggestions:

introduction section: please incorporate paragraphs 1 and 2 to shorten the introduction section and make the background information more relevant. line 57-59 can be deleted because it is not contributing much to the flow of writing. Edited

methodology section: 1. Please mention the sample size calculation and sampling technique in detail (provide formula). According to the type 1 error value and accuracy equal to 5%, as well as considering the value of 50% for the percentage of knee ligament damage (in order to estimate the maximum sample size), at least 384 people should be examined in this study. In this research, the researcher studies about 402 patient by Census sampling in which 285 patients had ACL injury and included for analysis.
2. Please mention whether or not the normality of the data set was tested? Yes did in SPSS.
3. Please justify the use of statistical test based on the normality of the data. Consult statistician. Checked.
4. Please revise the statement mentioned in line 85/86, as it is confusing that you enrolled 285 or 402 patients. Better to mention it in the result section with flow of participants. Edited
5. As it is a cross-sectional descriptive analytical study, providing confidence intervals would be better.
6. You have mentioned that you included patients with knee complaints with pain, giving away, and locking. Did that mean all patients had locking symptoms? please clarify. No, any of the symptoms could be a presenting symptoms.


Result section: 1. please avoid explanation of the study findings. for e.g. significantly lower. Please provide study findings as it is and explain them in the discussion section. All the tables are self-explanatory, so, limited writing is sufficient. In addition, please provide which test was done to calculate p value in all 4 table legends. - Chi-squared test was done to calculate p value

Discussion section: it is weak and can be improved significantly by careful writing and direct comparison with previous study findings. Edited :
1. Please follow simple rule of academic writing that 1 paragraph should evaluate 1 outcome. Use format of breaking sentence, data, evaluation, analysis with relevant previous study findings, reflection (if applicable), and closing sentence.
2. Please do not create new information which was not mentioned in the objective and methodology. for eg., correlation (in line 194), I did not find anywhere it was mentioned. It is mentioned in Table 1 and paragraph 1 in result.
3. please delete line 156-164 as these statements are general remarks. Can be used in introduction section though.
4. please compare and contrast your findings with specific studies rather than in general with multiple citations.

conclusion: please delete line 213-215, as it is not in the scope of the study. Instead, it would be better to write the implications of the findings. for eg. such prevalence of ACL injury would..... .edited.

**Alireza Rahimnia**

**Regards,**

September 27, 2023
