# Supplementary material for: Prevalence and mechanisms of anterior cruciate ligament tears in military personnel: A cross-sectional study in Iran
Source: PLoS One. 2024 Jun 21;19(6):e0303326. doi: 10.1371/journal.pone.0303326 (PMC11192309; doi:10.1371/journal.pone.0303326)
Supplement: Attachment — Submitted filename: Response to Reviewers.docx [file pone.0303326.s004.docx]

**Dear Editor-in-chief**

Thank you for evaluating my work. All the helpful comments were reviewed and edited in the text as follows:

The reference list is correct and in sync with the text.
Lines 21-23. The meaning of the first sentence of the abstract is not clear. Please rephrase it. It is checked and revised.
Line 90. Please, move the approval number after the name of the Institution providing the approval. Checked , edited.
Line 96. There are two dashes which should be removed. Edited.
Line 121. Please change ; with . changed.
Line 125. Please change ; with . changed
Line 149. Remove one of the dashes. Removed.
Line 254. In addition. Edited.
Line 263. Since you did not assess and reported other kind of injuries, I suggest to change in “…the ACL injury is one the most common knee injuries.” Edited.

**Regards,**

**Monday, April 22, 2024**
